# Can We Routinely Employ the Use of Low-Pressure Gynaecological Laparoscopy? A Systematic Review

**DOI:** 10.7759/cureus.15348

**Published:** 2021-05-31

**Authors:** Jack Hamer, Edward Jones, Amy Chan, Farshad Tahmasebi

**Affiliations:** 1 Obstetrics and Gynaecology, Russells Hall Hospital, The Dudley Group National Health Service (NHS) Foundation Trust, Dudley, GBR; 2 Anaesthesiology, Russells Hall Hospital, The Dudley Group National Health Service (NHS) Foundation Trust, Dudley, GBR

**Keywords:** minimally invasive laparoscopy, gynae, pneumoperitoneum, low-pressure, postoperative pain

## Abstract

Clinicians have learnt valuable lessons throughout the COV-SARS-2 pandemic, many of which have produced solutions that we aim to continue to implement within the foreseeable future. Optimising patients’ surgical pathways to reduce the length of stay and complications is an area of particular importance, both for maximal utilisation of available resources and for reduction of the exposure of inpatient and elective patients to an increased risk of infection within healthcare facilities.

The aim of this review was to investigate the possible implications of using low-pressure laparoscopic gynaecological surgery versus standard- or high-pressure pneumoperitoneum surgeries. The primary outcome was postoperative pain, with secondary outcomes including duration of surgery, length of inpatient stay and rate of complications.

MEDLINE, Embase and Cochrane CENTRAL were searched from inception to December 2020. We searched for published randomised control trials comparing low-pressure laparoscopic surgery (≤8 mmHg) to at least one additional standardised pneumoperitoneum pressure (≥12 mmHg and/or ≥15 mmHg). A total of 203 studies were reviewed, five of which were included in this analysis. Studies comparing low-pressure laparoscopic surgery against gasless abdominal cavities were excluded.

The meta-analysis of the results was pooled and calculated within RevMan 5.0 software (Cochrane, London, England). Studies using a visual analogue scale (1-10) to compare low versus standard pneumoperitoneum pressures did not display a significant diminution of postoperative pain at ≤ 6 or 24 hours: -0.30 [95% CI -0.63, 0.03] and -0.66 [95% CI -1.35, 0.02], respectively. Studies additionally demonstrated worse visualisation of the surgical field within the low-pressure group (risk ratio 10.31; 95% CI, 1.29-82.38 I^2^ = 0%). Studies measuring postoperative pain using a numerical rating scale displayed significant pain reduction at all hours measured (p ≤ 0.01). The rate of intraoperative complications was 1% for all groups measured. Cumulative analysis of the duration of surgery did not differ significantly between groups (p = 0.99).

The pandemic has revealed new issues that must be addressed by clinicians to promote the safety of patients and the efficiency of inpatient stay. This review has paved the way for new possibilities and innovative approaches to address the issue of optimising patient surgical pathways; however, at present, we cannot give a firm justification for the use of low-pressure gynaecological laparoscopy. Reasons for this include the minimal reduction in pain scores between low, standard and high pneumoperitoneum pressures, leading to a mixture of statistically significant results, as well as a reduction in the visualisation of the surgical field and the small population sizes in the reviewed papers. Additional research is required to further explore the potential clinical benefits of gynaecological laparoscopy to ensure its effective ambulatory use within mainstream surgical operations.

## Introduction and background

During the SARS-COV-2 pandemic, hospitals have been under extreme pressure, with their capacity having stretched beyond its limits. The pandemic has notably caused problems with inpatient hospital flow, leading to the stagnation of patients within wards. This poses an increased risk of the development of hospital-acquired infections [1].

Clinicians have learnt valuable lessons throughout the duration of the pandemic, many of which have produced solutions that we aim to continue to implement in the foreseeable future. Optimising patients’ surgical pathways to reduce the length of stay and complications is an area of particular importance both for maximal utilisation of available resources and for the reduction of the exposure of inpatient and elective patients to an increased risk of infection within healthcare facilities [2].

Laparoscopic surgery, particularly within gynaecological procedures, is frequently the most favourable surgical procedure, compared to open surgery. Laparoscopic surgery provides well-known advantages, including earlier patient mobilisation, reduced length of stay and reduced pain, alongside a decreased incidence of surgical site infections [3,4].

The Royal College of Obstetricians and Gynaecologists (RCOG) recommends that intra-abdominal pressure should be maintained between 12 and 15 mmHg to sustain a suitable surgical field of view, as well as to minimise ventilatory and haemodynamic risks to the patient [5-7]. However, a hypercapnic intra-abdominal environment can lead to the overt stimulation of the sympathetic nervous system, resulting in cases of postoperative shoulder tip and abdominal pain that may require opioid relief [8-12]. This has been noted in the previous literature to occur in up to 70% of patients 24 hours after gynaecological surgery, which can subsequently impede patient discharge on a timely basis until the pain has been adequately managed [13].

In recent years, emerging research has tackled this issue by exploring the use of lower pneumoperitoneum pressures (≤8 mmHg) during surgical procedures. This research may be significant in the streamlining of lessons learned from the pandemic. Interestingly, Vlot et al. highlighted in 2013 that 85% of abdominal expansion during porcine laparoscopic surgery was achieved at a pneumoperitoneum pressure of 10 mmHg [14]. A pertinent area of evolving research, therefore, is how to achieve maximal expansion of the abdomen while minimising insufflation pressures. This may help to marry the reduction of postoperative pain, the reduction of intra-operative blood loss and a sustained surgical field of view [15].

This systematic review thus delves into the literature available on low-pressure gynaecological laparoscopy versus standard and, in some cases, high pneumoperitoneum pressures, while evaluating both intra- and postoperative outcomes.

## Review

Method

The principles and results of this review adhered to the Preferred Reporting Items for Systematic Reviews and Meta-Analysis (PRISMA) guidelines [16].

Search Strategy

The electronic databases MEDLINE, Embase and the Cochrane library were used to search all available studies from inception to December 2020 and to analyse the available material on postoperative pain within low-pressure laparoscopy. The keywords and index terms used included gynaecological laparoscopy, gynaecologic laparoscopy, laparoscopic hysterectomy, low pressure, pneumoperitoneum and insufflation. To increase search accuracy, a population, intervention, comparison and outcome (PICO) model was used, as follows: Patient: women, Intervention: low-pressure gynaecological laparoscopy, Comparison: standard-pressure gynaecological laparoscopy, Outcome: postoperative pain. The details of this search strategy can be found within the appendices under Tables 4-6.

Inclusion and Exclusion Criteria

Only texts published in the English language were included. All records deemed eligible for assessment had their reference lists scrutinized to identify any additional citations to be included in the analysis that may not otherwise be extracted through the standardised literature search. One additional study was retrieved in this manner.

Studies including multiple laparoscopy insufflation pressures were included; however, all records eligible for qualitative and quantitative analysis were required to include at least a comparison between a low-pressure and standard-pressure pneumoperitoneum. Studies that included both benign and malignant operations were included in this review.

All studies analysed were required to include outcomes on postoperative pain; however, pain could be measured using different standardised methods. Differences were assessed on the basis of the overall mean pain scores between laparoscopy pressures and times assessed postoperatively. Our primary aim was to measure postoperative pain; secondary outcomes included estimated blood loss, duration of surgery, types of operations and rate of complications. An additional factor measured included the number of surgeons operating. This was deemed to be a relevant factor, as the inclusion of multiple surgeons within one study would aim to reduce operator bias, thus allowing the results to be more generalisable to the surgical population.

All records of patients under the age of 18 were excluded. Additionally, studies measuring standard pneumoperitoneum pressures versus gasless abdomens were excluded. Cohort, case-control and case report studies were excluded, as was low-pressure non-gynaecological laparoscopy. Studies excluded based on full-text analysis can be found within the appendices under Table 7.

Data Extraction

Two independent authors screened eligible studies by title, abstract and, when required, full text to see if they met the inclusion criteria for review. Any disagreements were concluded through arbitration with the senior author. For each trial assessed, information was collected regarding the study design, characteristics of participants, interventions used, comparisons made and postoperative outcomes assessed. Low-pressure insufflation pressures were set at ≤8 mmHg, standard insufflation pressures were set between 12 and 15 mmHg, and high insufflation pressures were set at ≥15 mmHg.

For studies measuring postoperative pain on a visual analogue scale, the mean differences, standard deviations and 95% confidence intervals were pooled and calculated. Consistency I^2^ measurements to determine heterogeneity calculated using RevMan5 software (version 5.3) (Cochrane, London, England) [17]. Subgroup analysis was performed to detect variance in heterogenicity.

Analysis of Study Quality

**Figure 1:**
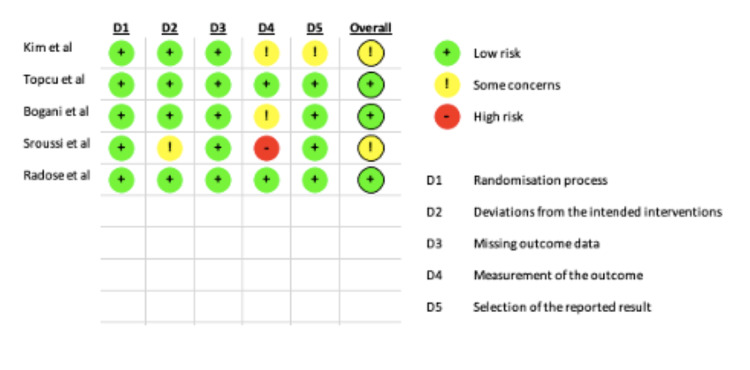
Risk of bias analysis for included studies.

This review assessed five randomised control trials for quality using the risk of bias analysis tool created by the Cochrane Collaboration [18]. Analysis of bias was assessed under five main domains: randomisation process, deviations from the intended interventions, missing outcome data, measurement of the outcome and selection of the reported result. Each study has an overall grade for the evaluation of bias. An overall analysis of quality assessment can be found in Figure 1.

Results

**Figure 2:**
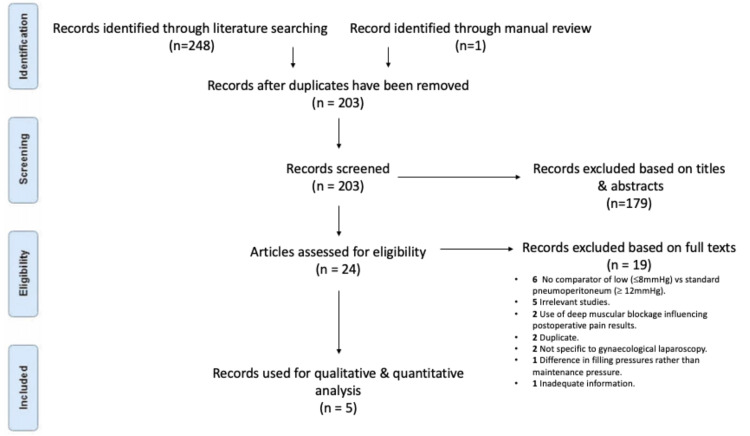
Identification of records used.

A total of 203 studies were identified for possible inclusion after duplicates were removed. Of these, 179 studies did not meet the eligibility criteria based on their titles and abstract content. Through further analysis, 19 of the 24 remaining studies were found not to meet the criteria for analysis after full-text assessment. Finally, five studies were selected for discussion. Figure 2 displays the process used to retrieve accurate records.

Five studies for a total of 380 patients were included in this review. These studies analysed pain, mainly shoulder tip and abdominal pain, up to 24-48 hours postoperatively. The characteristics of all included studies can be seen in Table 1.

**Table 1:** Characteristics of included studies. Index: BMI = body mass index, BSO = bilateral salpingo-oophorectomy, Lap = Laparoscopic, No. = number, LP = low pressure, SP = standard pressure, HP = high pressure, LASH = laparoscopic supracervical hysterectomy, TLH = total laparoscopic hysterectomy, USO = unilateral salpingo-oophorectomy, TL = tubal ligation, TAH – total abdominal hysterectomy, LN = lymph node, TLH, Total laparoscopic hysterectomy, LASH = Laparoscopic supracervical hysterectomy, EBL = estimated blood loss, NO = number.

Paper	Year	Age of patients	No. of patients	BMI	Type of procedures performed	Length of Procedure/s (min)	Number of surgical teams	EBL (mL)
Kim et al. [19]	2006	LP: 47.3 ± 10.1	LP: 23	LP: 23.8 ± 3.2	Ovarian cystectomy USO or BSO TAH ± BSO Staging laparoscopy Myomectomy and radical hysterectomy with LN dissections	LP: 148 ± 74.8	One	LP: 133.9 ± 54.7
		SP: 41.4 ± 9.7	SP: 23	SP: 23.5 ± 3.1		SP: 128 ± 64.0		SP: 54.1 ± 34.3
Topcu et al. [20]	2014	LP: 34.6 ± 7.2	LP: 54	LP: 25.2 ± 4.2	Tubal ligation Cystectomy TL+ cystectomy Diagnostic laparoscopy Salpingectomy	LP: 42.3 ± 20.9	One	LP: 31.0 ± 44.8
		SP: 33.1 ± 6.6	SP: 45	SP: 24.9 ± 4.9		SP: 24.0 ± 10.7		SP: 19.5 ± 20.1
		HP: 34.0 ± 6.8	HP: 51	HP: 25.3 ± 3.9		HP: 23.5± 10.1		HP: 12.3 ± 13.0
Bogani et al. [21]	2014	LP: 47 ± 6	LP: 20	LP: 24.9 ± 5.1	Lap hysterectomy	LP: 58 ± 9.7	One	LP: 77.5 ± 83.4
		SP: 49 ± 9.9	SP: 23	SP: 25.4 ± 6.9		SP: 64.4 ± 30		SP: 93.1 ± 96.7
Sroussi et al. [22]	2017	LP: 33 (19-45)	LP: 30	LP: 22.1 (19-29.4)	Diagnostic Lap Extra-uterine pregnancy Tubal surgery Cystectomy Salpingectomy Myomectomy	LP: 26 (9-83)	Two	/
		SP: 35 (25-58)	SP: 30	SP: 22.6 (19.3-31.2)		SP: 30 (10-85)		/
Radosa et al. [23]	2019	LP: 51 (40–79)	LP: 91	LP: 26 (21.6–35.9)	TLH, LASH	LP: 95 (51–202)	Four	/
		SP: 49 (39–78)	SP: 87	SP: 27.8 (19–29.2)		SP: 120 (50–249)		/

Studies Implementing a Visual Analogue Scale

Topcu et al. performed a prospective randomized trial of 150 patients undergoing laparoscopic gynaecological surgery, who were split into three laparoscopic categories: low pressure (8 mmHg), standard pressure (12 mmHg) and high pressure (15 mmHg) [20]. Indications for surgery included cystectomy, tubal ligation and salpingectomy. Postoperative pain was measured on a visual analogue scale ranked from 0 to 10 at hours 6, 12 and 24 postoperatively. Postoperative pain was experienced by fewer patients at six hours (p = 0.07) and significantly less at 12 hours for patients undergoing low-pressure laparoscopy, compared to those undergoing standard- and high-pressure (p < 0.001) pneumoperitoneum [20]. Low and standard insufflation pressures were not significantly different between each group at 24 hours postoperatively; however, in comparison to high-pressure groups, statistical significance was achieved (p < 0.001) [20]. Conversely, mean operative time was significantly higher within the low-pressure group than in the other groups, while intraoperative blood loss was statistically higher in the low-pressure group than in the high-pressure group. Duration of inpatient stay did not differ between the groups.

In conjunction, Bogani et al. explored the implementation of low-pressure laparoscopy (8 mmHg) versus standard pneumoperitoneum pressure (12 mmHg) in 42 patients undergoing laparoscopic hysterectomies [21]. Indications for surgery included uterine myoma, cervical dysplasia and endometrial hyperplasia. The pain was measured on a 100-mm visual analogue scale at hours 1, 3 and 24 postoperatively [21]. Abdominal pain did not differ significantly at hours 1, 3 and 24 (p = 0.37, p = 0.69 and p = 0.19, respectively). On the contrary, shoulder tip pain was experienced significantly less at hours 1 and 3 postoperatively within the low-pressure group, however not within hours 24 (p = 0.01, p = 0.03 and p > 0.05, respectively). Additionally, estimated blood loss, duration of surgery and length of hospital inpatient stay did not differ significantly (p = 0.36, p = 0.80 and p = 0.17, respectively) [21]. No intra-operative complications or conversion to open surgery were reported in the low-pressure group, whereas one complication occurred within the standard-pressure group. Furthermore, the use of rescue analgesics was lower in the low-pressure group (20%) than in the standard pressure group (41%).

Table 2 presents the summarised mean postoperative pain scores for all time categories measured using a visual analogue scale, including standard deviations and significance scores.

**Table 2:** Summary of papers measuring post-operative pain with a visual analogue scale. Index: a = low-pressure group is significantly different from a standard pressure group, b = low-pressure group is significantly different from high-pressure group, c = low-pressure group is significantly different from high-pressure group, d = standard pressure group is significantly different from high-pressure group, hr = hour.

Paper	Total number of patients	Low-pressure score with standard deviation		Standard pressure score with standard deviation		High-pressure score with standard deviation		P-value
Topcu et al. [20]	150	≤6 hr	4.83 ± 1.89	≤6 hr	5.51 ± 1.75	≤6 hr	5.62 ± 1.23	0.07
		12 hr	2.31 ± 1.68^a, b^	12 hr	3.08 ± 1.54	12 hr	3.57 ± 0.86	<0.001
		24 hr	1.07 ± 1.38^c^	24 hr	2.41 ± 1.33^d^	24 hr	2.41 ± 0.45	<0.001
Bogani et al. [21]	42	≤6 hr	1.85 ± 1.63	≤6 hr	2.09 ± 1.82	≤6 hr	N/A	<0.05
		24hr	0.44 ± 0.89	24 hr	0.68 ±0.82	24 hr	N/A	>0.05

Cumulative analysis of the two studies demonstrated worse visualisation of the surgical field within the low-pressure group (risk ratio 10.31; 95% CI, 1.29-82.38 I^2^ = 0%).

**Figure 3:**
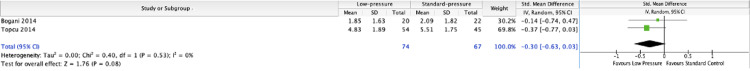
Post-operative pain: Low-pressure vs standard-pressure at ≤6 hours. Index: CI = confidence interval, SD = standard deviation, Std = standardised.

**Figure 4:**
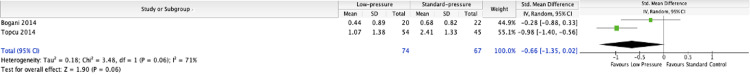
Post-operative pain: Low-pressure vs Standard-pressure at 24 hours. Index: CI = confidence interval, SD = standard deviation, Std = standardised.

Figures 3 and 4 present a comparative meta-analysis of low and standard pressure at hours 6 and 24 postoperatively with respect to postoperative pain.

Studies Implementing a Numerical Analogue Scale

Radosa et al. scrutinize further by blinding 87 patients within the low-pressure group (8 mmHg) and 91 patients within the standard-pressure group (12 mmHg) and exploring their symptoms up to 48 hours postoperatively, using a numerical analogue scale instead of a visual analogue scale [23]. The main indications for surgery included symptomatic uterine fibroids, endometriosis and descensus uteri. Postoperative shoulder tip and abdominal pain were measured as a primary outcome for all groups at hours 3, 24 and 48. Abdominal pain and shoulder tip pain were experienced by far fewer patients in all-time categories in the low-pressure group (p < 0.001) [23]. Interestingly, patients in the low-pressure group additionally experienced fewer episodes of fatigue and bloating at all postoperative times recorded (p < 0.01) [23]. Body mass index and duration of surgery did not differ significantly between pressure groups. No intraoperative complications occurred within the low-pressure group; however, two intraoperative bladder injuries arose within the standard-pressure group (p = 0.15) [23]. A single postoperative complication of vaginal bleeding was noted within the low-pressure group, requiring no intervention, compared to three postoperative complications within the standard-pressure group, one of which required a second laparoscopy for vaginal cuff dehiscence.

A 2017 pilot study by Sroussi et al. delved into this topic further by exploring the possibility of performing laparoscopic surgery at even lower pneumoperitoneum pressures (7 mmHg) than in previous literature with the use of the AirSeal® system. Thirty patients underwent gynaecological laparoscopy at pressures of 7 mmHg, and 30 further patients at 15 mmHg, for benign laparoscopic conditions [22]. As in the study by Radosa et al., postoperative shoulder tip pain was measured on a numerical rating scale. The pain was measured at hours 4, 8 and 24 [22]. No complications or conversion to open surgery were reported within the low-pressure group, and the incidence of shoulder pain was significantly lower in all-time groups measured compared to standard pneumoperitoneum pressures (overall p = 0.003). The length of the procedure did not significantly differ between groups (p = 0.55), and 47% of the low-pressure study group felt as if they could be discharged on the same day, as opposed to 23% of the standard pressure group.

Table 3 presents the summarised mean postoperative pain scores for all time categories measured using a numerical analogue scale, including standard deviations and significance scores.

**Table 3:** Summary of papers measuring post-operative pain with a numerical analogue scale.

Paper	Total number of patients	Low-pressure score		Standard pressure score		P-value
		Hours	Mean (range)	Hours	Mean (range)	
Radosa et al. [23]	178	≤12	1 (0-5)	≤12	3(0-10)	≤0.01
		24	1(0-8)	24	3(0-9)	≤0.01
		48	1(0-4)	48	1(0-8)	≤0.01
Sroussi et al. [22]	60	≤12	0.8 (0-7)	≤12	2.4 (0-9)	P=0.003
		24	0.5(0-6)	24	1.5(0-6)	P=0.004

Study Measuring Pain as Secondary Outcome

In contrast, Kim et al. randomised 46 patients into low-pressure (8 mmHg) and standard insufflation pressure (13 mmHg) groups [19]. Indications for laparoscopic surgery included total hysterectomy, staging laparoscopy, and unilateral and bilateral salpingo-oophorectomy. The primary outcome for this study was to explore rates of postoperative nausea and vomiting, and the secondary outcomes included the consumption of analgesics and duration of surgery [19]. Kim et al. found no significant difference in the consumption of analgesics between study groups at all times measured (2, 6 and 24-hours postoperatively) [19]. The study did, however, highlight that low-pressure laparoscopy was associated with a significantly greater volume of blood loss (p < 0.05) [19]. No complications or conversions to open surgery were reported in either group. Finally, overall postoperative nausea and vomiting did not differ significantly between the groups (p = 0.31).

Discussion

Within this review, we found that all studies but one demonstrated at least a single reduction in postoperative pain by low-pressure laparoscopic surgery (≤8 mmHg) compared to standard-pressure laparoscopy (≥12 mmHg) at the postoperative times measured. However, Bogani et al. favoured follow-up times less than 12 hours postoperatively, in contrast to Topcu et al., who chose times significantly above 12 hours postoperatively, despite using the same mode of measurement [20,21]. The included studies additionally demonstrated that low-pressure pneumoperitoneum was associated with a poorer visualisation of the surgical field. Overall, most included studies were in agreement that no groups differed significantly regarding the duration of surgery, duration of inpatient stay, rate of complications, failure rate and blood loss; however, disagreements regarding secondary outcomes were clearly demonstrated in particular selected studies.

We observed a reduction in postoperative pain in low-pressure groups in studies using a visual analogue scale, but these results were not significant when particular studies using this analogue scale were combined within a meta-analysis. A significant reduction of pain in low-pressure groups was observed in all-time categories by studies measuring with a numerical analogue scale, but it was not feasible to conduct a meta-analysis to truly evaluate the results. Despite significant results, any reduction in pain scores between groups was minimal, and clinical descriptions of relative pain from severe to moderate, moderate to mild or mild to milder were not evaluated further within included studies, thus making it difficult to assess the results to determine the true reduction in pain between groups based solely on numerical values. Studies additionally displayed little difference in the duration of hospital stay and consumption of analgesics between groups, thus further reducing the amplitude of significance for the interpretation of pain scores alone.

The results of our review are in concordance with those of previous reviews by Hua et al. and Raval et al., who also explored the effect of low-pressure laparoscopy cholecystectomies on postoperative pain [24,25]. In their studies, low-pressure laparoscopic surgery was found to be associated with reduced postoperative pain scores and a reduced inpatient stay. The latter finding was not apparent within our review, but the current published material on gynaecological procedures is less prevalent than the literature on other surgical specialities.

**Figure 5:**
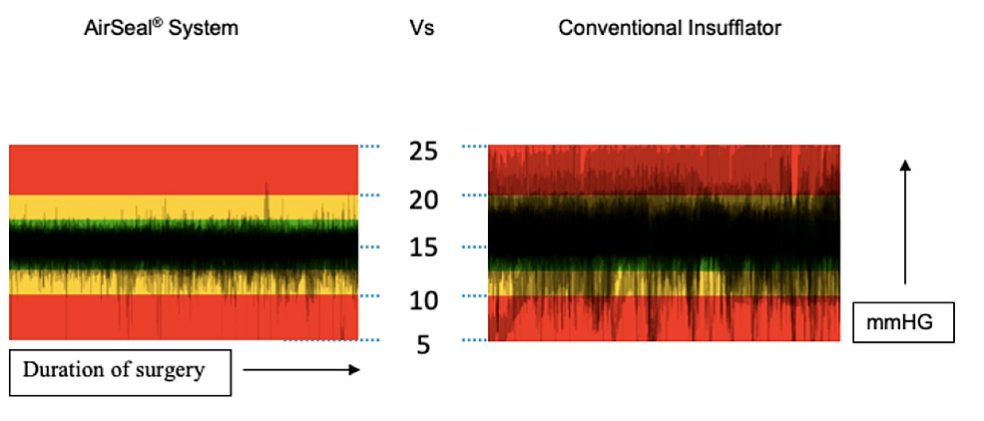
Stability of pneumoperitoneum between insufflator systems. Index: mmHG = millimetre of mercury.

Confounding variables to which could be attributed the fluctuating nature of reported pain scores are the initial trocar insertion pressure, time taken to deflate the abdomen to lower pressures, the irregular amplitude of intra-abdominal pressures during each procedure once sustained low or standard pneumoperitoneum pressure was achieved and, finally, degree of Trendelenburg tilt. Insufflator systems can control regulatory intra-abdominal pressures and flow rates; however, the rate of response can vary according to manufacture specifications [26,27]. One study in this review explored low-pressure laparoscopy with the use of Airseal®. Real-time pressure detection and immediate adjustment with this insufflator system have been shown to maintain a stable pneumoperitoneum even at low pressures [28]. Bucur et al. revealed that the stability preserved with the Airseal® is greater compared to a standard insufflator system [29]. A visual schematic is displayed in Figure 5. Future studies on the application of Airseal® to aid in the reduction of possible confounding variables may be warranted.

 

In this review, we observed a low rate of complications and conversion to open surgery within low-pressure groups. However, the studies reviewed performed most surgical procedures for benign conditions; thus, increasing the use of low-pressure procedures for malignant conditions may demonstrate shortfalls in low-pressure, particularly when it is combined with a reduction in visual field. Furthermore, studies within this review contained low population sizes. As gynaecological complications tend to be uncommon in surgical procedures, future studies may warrant larger population sizes to detect any differences in complication rates between pressure groups. Overall, we believe that this review does not contain adequate statistical evidence to convincingly promote the use of low-pressure laparoscopic surgery.

Limitations

This review included five papers, consisting of a total of 380 participants. Although these studies provide novel insights into a previously unexplored area of research, the limited variety of papers exploring low-pressure gynaecological laparoscopy make it difficult to draw sufficient conclusions for application within the medical community. Many studies in this review did not measure pain further than 24 hours postoperatively. Future studies may consider measuring pain for longer than 24 hours to provide consistency in published results, making them suitable for analysis to identify any further benefits of low-pressure laparoscopy.

Second, whilst the included studies evaluated pain on a numerical value, the studies did little to evaluate the clinical impact of pain. Albeit the differences in pain scores might have been significant at times, the included studies did little to evaluate the use of additional important outcomes such as prolonged narcotic use, delayed discharge or delayed mobility. Future studies may warrant a greater exploration of these outcomes, as a sole decrease in post-operative pain would not be substantial enough to employ the use of ambulatory low-pressure laparoscopy in routine practice.

Furthermore, 60% of the included studies had only one surgical team performing all operations on participants. This may indicate an element of operator bias, as surgeons within the studies may be more experienced than the general cohort in performing low-pressure gynaecological operations, thus reducing the observed surgical failure rates and intra-abdominal complications, such as expected blood loss. A more diverse surgical team within future studies will help provide more consistent results that are more representative of the wider surgical community. If low-pressure laparoscopy were to be incorporated as an ambulatory approach to gynaecological operations, the justifications for this need to be explored much further.

Despite these limitations, our study rigorously explored major databases to retrieve all available material and conduct a thorough analysis of this topic. We included a variety of randomised control trials comparing at least two surgical pneumoperitoneum pressures without regard to the date of publication or type of surgeries performed to enhance the validity and generalisability of the results.

## Conclusions

To conclude, this review cannot justify the current use of low-pressure gynaecological laparoscopy. Meta-analysis of studies using the visual analogue scale did not display a significant reduction in post-operative pain. However, studies using the numerical analogue scale did display significant results in all times measured, but a meta-analysis could not be conducted. Studies displayed worse visualisation of the abdominal cavity. The rate of secondary complications, duration of inpatient stay, estimated blood loss and duration of surgery were relatively similar between pressure groups. The studies included in this review were of sound quality and displayed a low risk of bias; thus, we conclude that this review is of a moderate to the high level of evidentiary quality.

Following the resolving SARS-COV-2 pandemic, the medical community aims to harness the lessons learned regarding hospital inpatient management and infection control. Optimising surgical pathways can aid in streamlining patients to discharge in a timelier manner. Low-pressure laparoscopy may assist with this in the near future; however, the beneficial qualities needed to substantiate its use in routine gynaecological procedures are not currently proven. Additional research will be needed before employing low-pressure genealogical surgery as an ambulatory modality for surgeons.

## Appendices

**Table 4:** Search strategy on MEDLINE.

Search term	Results
exp laparoscopy/	100,686
Low pressure.tw.	6,258
Gynaecological laparoscopy.tw.	151
Gynaecologic laparoscopy.tw.	35
Low vs standard.tw.	22
1 or 2 or 3 or 4 or 5	106,795
exp pneumoperitoneum/	3,838
exp insufflation/	3,449
exp pressure/	114,892
7 or 8 or 9	121,024
exp pain/	404,650
Post-operative.tw.	50,655
Postoperative pain.tw.	21,824
11 or 12 or 13	457,209
6 and 10 and 14	174

**Table 5:** Search strategy on Embase.

Search term	Results
exp laparoscopy/	167,712
Low pressure.tw.	10,876
Gynaecological laparoscopy.tw.	229
Gynaecologic laparoscopy.tw.	62
Low vs standard.tw.	42
1 or 2 or 3 or 4 or 5	178,408
exp pneumoperitoneum/	10,750
exp insufflation/	13,610
exp pressure/	97,784
7 or 8 or 9	120,945
exp pain/	1,379,115
Post-operative.tw.	125,665
Postoperative pain.tw.	36,885
11 or 12 or 13	1,489,266
6 and 10 and 14	1,599

**Table 6:** Search strategy on Cochrane.

Search Term	Results
exp laparoscopy/	8,345
Low pressure.tw.	26,363
Gynaecological laparoscopy.tw.	828
Gynaecologic laparoscopy.tw.	1,079
Low vs standard.tw.	9,011
1 or 2 or 3 or 4 or 5	41,486
exp pneumoperitoneum/	1,468
exp insufflation/	1,704
exp pressure/	151,950
7 or 8 or 9	153,392
exp pain/	191,676
Post-operative.tw.	50,655
Postoperative pain.tw.	21,824
11 or 12 or 13	457,209
6 and 10 and 14	174

**Table 7:** Studies excluded within this review.

Study title	Authors	Year
Interventions to reduce shoulder pain following gynaecological laparoscopic procedures	Kaloo et al.	2019
Pain after laparoscopic surgery: Focus on shoulder-tip pain after gynecological laparoscopic surgery.	Sao et al.	2019
Effects of deep versus moderate neuromuscular blockade in laparoscopic gynecologic surgery on postoperative pain and surgical conditions: protocol for a randomized controlled trial.	Robertis et al.	2018
The impact of carbon dioxide pneumoperitoneum on ovarian ischemia-reperfusion injury during laparoscopic surgery: a preliminary study.	Akdemir et al.	2018
Association between intraabdominal pressure during gynaecologic laparoscopy and postoperative pain.	Kundu et al	2017
A randomized double-blind controlled trial of different filling pressures in operative outpatient hysteroscopy.	Haggag et al.	2017
Reply to: postoperative shoulder pain after laparoscopic hysterectomy with deep neuromuscular blockade and low-pressure pneumoperitoneum.	Madsen et al.	2017
Deep neuromuscular blockade and low insufflation pressure during laparoscopic hysterectomy.	Madsen et al.	2017
Postoperative shoulder pain after laparoscopic hysterectomy with deep neuromuscular blockade and low-pressure pneumoperitoneum: A randomised controlled trial.	Madsen et al.	2016
What is the evidence for the use of low-pressure pneumoperitoneum? A systematic review.	Brunschot et al.	2016
Insufflation with humidified and heated carbon dioxide in short-term laparoscopy: a double-blinded randomized controlled trial.	Herrmann et al	2015
Impact of intraperitoneal pressure of a CO2 pneumoperitoneum on the surgical peritoneal environment.	Matsuzaki et al.	2012
Factors associated with pain following operative laparoscopy: a prospective observational study.	Healey et al.	2009
Diastolic function: the influence of pneumoperitoneum and Trendelenburg positioning during laparoscopic hysterectomy.	Russo et al.	2009
Laparoscopic entry: a review of techniques, technologies, and complications.	Vilos et al.	2007
A prospective randomised double-blind placebo controlled trial to assess whether gas drains reduce shoulder pain following gynaecological laparoscopy.	Swift et al.	2004
Warming of insufflation gas during laparoscopic hysterectomy: effect on body temperature and the autonomic nervous system.	Nelskyla et al.	1999
Gasless laparoscopic gynecologic surgery.	Ercole et al.	1996
Haemodynamic changes due to trendelenburg positioning and pneumoperitoneum during laparoscopic hysterectomy.	Hirvonen et al.	1995
